# Therapeutic imprinting of the immune system: towards a remission of AIDS in primates?

**DOI:** 10.1186/1742-4690-9-75

**Published:** 2012-09-14

**Authors:** Andrea Savarino, Enrico Garaci

**Affiliations:** 1 Department of Infectious, Parasitic and Immune-mediated Diseases, Istituto Superiore di Sanità, Viale Regina Elena, Rome 299 00161, Italy; 2 Istituto Superiore di Sanità, Viale Regina Elena, Rome, Italy

**Keywords:** Macaque AIDS model, Functional cure, Antiretroviral therapy, Central memory CD4^+^ T-cells, Maraviroc, Auranofin, Buthionine sulfoximine (BSO), Anti-reservoir

## Abstract

Our inability to cure HIV/AIDS is related to the ability of the virus to establish reservoirs during treatment. In order to develop new strategies, it is certainly essential that a suitable animal model be implemented. In the recent work of Shytaj et al., it has been possible to inhibit viral replication to levels below the assay detection limit in the macaque AIDS model. Moreover, different therapeutic regimens applied to the rhesus macaque AIDS model (herein reviewed), including ours, are starting to show the potential to induce, following therapy suspension, conditions reminiscent of a drug-free control of the infection.

## Introduction

**Figure 1:**
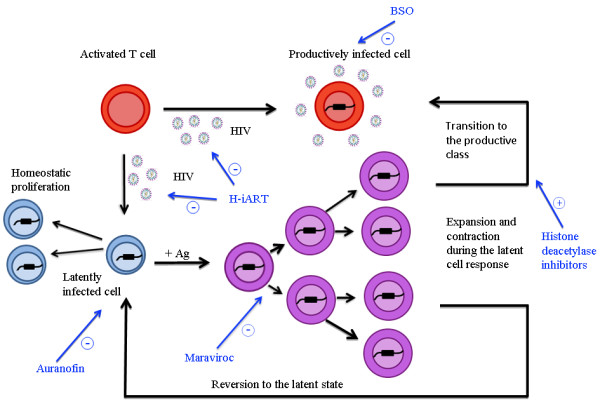
**Schematic representation of the most likely viral reservoir dynamics*in vivo*and the possible anti-reservoir drug strategies mentioned in the text.** Briefly, latently infected cells proliferate by either homeostasis or antigen-driven activation. Part of the activated cells becomes productively infected, spreading the virus to uninfected cells, while another portion either dies or reverts to a latent state. These phenomena contribute to the expansion of the viral reservoir during lentiviral infections. If cell-to-cell viral spread is blocked by highly-intensified antiretroviral therapy (H-iART), activation-driven proliferation and homeostatic proliferation represent the main mechanisms for the expansion/persistence of the viral reservoir during therapy. One important component of H-iART, i.e. the CCR5 blocker maraviroc, was shown in a recent study (Ref. [8]) also to decrease activation-induced proliferation of the central and transitional memory T-cells (encompassing the viral reservoir). Antilatency drugs such as the histone deacetylase inhibitors may favor the transition of the activated latently infected cells to the productive class [vulnerable to viral cytopathogenicity, specific immune responses, or pro-oxidant drugs auch as buthionine sulfoximine (BSO)] at the expense of reversion of the same cells to the latent state. Finally, the gold salt auranofin, by decreasing the lifespan of the central and transitional memory T-cells, restricts their potential to expand by both homeostatic and activation-driven proliferation. Adapted and modified from Ref. [14].

Antiretroviral therapy (ART; usually a combination of three drugs) is unable to completely eliminate the virus from the infected organism. When the detection limit of the standard assays (usually 50 copies of viral RNA/mL) is decreased, persistence of low-level viremia is observable in the majority of the individuals under ART [1]. Moreover, the eradication of the virus is hampered by the existence of long-lived viral reservoirs, (mainly central and transitional memory CD4^+^ T-cells), which harbor silent copies of proviral DNA that cannot be targeted by antiretroviral drugs or the immune system [2]. Mainstream experimental approaches for the elimination of the viral reservoir include stem cell transplantation to renovate the immune system [3], or the “shock-and-kill” strategies, i.e. 1) intensified ART (iART) with at least four drugs targeting viral replication at multiple steps, 2) induction of viral escape from latency through specific antilatency drugs such as the histone deacetylase inhibitors, and 3) elimination of the infected cells through either viral cytopathogenicity, the immune system or specific drugs [3,4] (Figure1). An alternative approach is aimed at decreasing the lifespan of the central and transitional memory T-cell pools [5] (Figure1). Non-human primate models are suitable experimental platforms for pre-clinical testing of investigational anti-reservoir drugs discovered in the laboratory [6]. Due to their close phylogenetic relationship with humans, macaques (*Macaca sp.*) infected with sooty mangabay-derived viruses (SIVmac), such as SIVmac251, represent interesting models for studying the pathophysiology of HIV/AIDS in humans; however, the paucity of antiretrovirals able to cross-inhibit SIVmac rendered particularly difficult maintaining an undetectable viremia mimicking the conditions of HIV-infected individuals under ART.

## Main text

The findings that SIVmac is susceptible to the nucleosidic/nucleotidic reverse transcriptase inhibitors emtricitabine and tenofovir [6] and to the integrase inhibitor raltegravir [7] were fundamental for the establishment of current macaque models for suppressive ART regimens that may also include the protease-inhibitor darunavir boosted with ritonavir (iART) [5]. These drug cocktails were shown to control viremia at undetectable levels for prolonged periods in macaques in the early chronic SIVmac251 infection [5]. In a recent study coordinated by our Institution, Shytaj et al. show that a five–drug regimen (tenofovir/emtricitabine/raltegravir/darunavir/r/maraviroc), dubbed highly-intensified ART (H-iART), can control viremia to undetectable levels in a wider array of macaques including, for the first time, those in pre-AIDS stage and those with high viral set points (> 5.5 *Log* viral RNA copies/mL) [8]. This model was also suitable for providing further insight into viral persistence during therapy. By lowering the viral detection limit in plasma to 3 RNA copies/mL, and by analyzing viral RNA in anatomical compartments such as the rectum and inguinal lymph nodes, no ongoing viral replication was detected in the majority of the treated macaques [8]. One unexpected finding was a likely decrease in the viral reservoir, as shown by the occurrence of a three-phase decay of viral DNA, with the third phase likely corresponding to the decay of latently infected cells. This observation was supported by a decrease in the viral set-point following therapy suspension. Potential anti-reservoir effects of the CCR5 blocker maraviroc (i.e., the crucial component of H-iART) were also observed in independent studies of Gutierrez et al. and Ananworanich et al. in humans [9,10]. The aforementioned study of Shytaj et al. provides the grounds for these results, showing that maraviroc decreases activation-driven proliferation of the central and transitional memory T-cells *in-vitro* (Figure1) [8]. These effects prompted evaluation of H-iART in combination with more specific anti-reservoir strategies. In a pilot study, animals were treated with H-iART and the gold-drug auranofin, which selectively kills the central and transitional memory-cells (encompassing the viral reservoir [5]). Following treatment suspension, these animals displayed very low viral set-points (≤ 10^3^ viral RNA copies/mL) upon treatment interruption, though remaining persistently viremic [8]. More surprising results, i.e. a drug-free control of viremia, were obtained by re-treating with a short H-iART cycle (≈ 1.5 months) at rebound (which mimics a novel acute infection-like condition, i.e. a new therapeutic window) [5,8] or by adding, to the HiART/auranofin combination, buthionine sulfoximine [11] (BSO, a drug previously shown to contribute to viral escape from latency and selectively kill productively infected cells [4]; see Figure1). Although safety issues will require further evaluation, the treatments were well tolerated by the macaques, thus suggesting that restriction/death of the memory T-cells is relatively safe.

Van Rompay et al. [12] recently showed similar findings, following an entirely different approach, i.e. treatment of rhesus macaques both in the acute and early chronic phase of SIVmac/RT-SHIV infection with a suboptimal and discontinued antiretroviral regimen consisting of tenofovir alone. Suspension of this treatment resulted in a “functional cure”, defined as “a condition in which virus is not eliminated but is controlled sufficiently by antiviral immune responses so that drug treatment can be withdrawn for prolonged periods of time” [12]. Both the results of our group and those of Van Rompay et al., though obtained with substantially different approaches that are not yet directly applicable to humans, open a window into a possible future scenario of a functional cure of AIDS. In both cases, upon treatment suspension, the virus attempts to escape the immune control but is consistently brought back to very low or undetectable levels and cannot reach a stably viremic set-point [8,11,12]. It will be interesting in the future to investigate on possible common mechanisms sparked by the different therapeutic approaches.

## Conclusions

The aforementioned studies show that a condition close to a functional cure, which had so far obtained only by treating during acute infection (as an example, see Ref [13]), is achievable pharmacologically in the chronic phase of SIV infection in rhesus macaques. Of note, these results have been obtained by following approaches that have so far been underestimated by the mainstream research for a cure of AIDS [3] and will therefore merit appropriate consideration for further mechanistic analysis, including mathematical modeling [14], subsequent formulation of candidate treatment protocols, and testing in larger numbers of macaques.

## Competing interests

The authors declare that they have no competing interests.

## Authors’ contributions

AS conceived the ideas described in the present viewpoint and drafted the manuscript. EG participated in the generation of the ideas presented in the manuscript. Both authors read and approved the final manuscript.
